# Supplementary material for: Curated and harmonised transcriptomics datasets of interstitial lung diseases
Source: Data Brief. 2025 Oct 14;63:112139. doi: 10.1016/j.dib.2025.112139 (PMC12581653; doi:10.1016/j.dib.2025.112139)
Supplement: Supplementary file 1 [file mmc1.zip › Supplementary_material/RNA-seq/fastQC reports/GSE184316/TRIMMED_SRR15926806_fastqc.html]

TRIMMED\_SRR15926806.fastq.gz FastQC Report 

FastQC Report

Sat 3 Dec 2022  
TRIMMED\_SRR15926806.fastq.gz

## Summary

- Basic Statistics
- Per base sequence quality
- Per sequence quality scores
- Per base sequence content
- Per sequence GC content
- Per base N content
- Sequence Length Distribution
- Sequence Duplication Levels
- Overrepresented sequences
- Adapter Content

## Basic Statistics

| Measure | Value |
| --- | --- |
| Filename | TRIMMED\_SRR15926806.fastq.gz |
| File type | Conventional base calls |
| Encoding | Sanger / Illumina 1.9 |
| Total Sequences | 9619257 |
| Sequences flagged as poor quality | 0 |
| Sequence length | 35-371 |
| %GC | 50 |

## Per base sequence quality

## Per sequence quality scores

## Per base sequence content

## Per sequence GC content

## Per base N content

## Sequence Length Distribution

## Sequence Duplication Levels

## Overrepresented sequences

| Sequence | Count | Percentage | Possible Source |
| --- | --- | --- | --- |
| AGGCTACACACTGAGGACTCTGTTCCTCCCCTTTCCGCCTAGGGGAAAGT | 169954 | 1.7668100561197189 | No Hit |
| GGGAAGCGGGGAATGTCTACGTGCGTATGCACGTGGCACTCTCTGCCCGA | 151589 | 1.5758909445916667 | No Hit |
| CAGTGGGGCCACGAGCTGAGTGCGTCCTGTCACTCCACTCCCATGTCCCT | 142656 | 1.4830251442497067 | No Hit |
| CACCTCCCCGAAGCTCAGGGAGAGCCCTGTTAGGGCCGCCTCTGGCCCTA | 129077 | 1.3418603952467432 | No Hit |
| CCCTGGCTGCTACAGGAGAATAGCAGACAGGTATAGTTAAGAAACAACTT | 59962 | 0.6233537579877532 | No Hit |
| AGCTGCTAAGTGCTGTGTTGTCGTTCCCCCTGCTTAAAATAAAGTTGTTT | 54633 | 0.567954468832676 | No Hit |
| CAATCCACTTGATCCCAACTCATCTCTCATTTATTTCGGCTTCTTTTATT | 48154 | 0.5005999943654691 | No Hit |
| AGACATCAAATCCTGCAGACAAGGGGAGCCCTCAGTCTGCAGGGCTCCAT | 47594 | 0.4947783389091278 | No Hit |
| TGATATGGTTCCTGGCAAGCCCATGTGTGTTGAGAGCTTCTCAGACTATC | 44777 | 0.4654933328010677 | No Hit |
| TGATGACACCCACCGCAACTGTCTGTCTCATATCACGAACAGCAAAGCGA | 44706 | 0.4647552300557101 | No Hit |
| CAGCAGTTTACACCTGCTAGGGTGTTCAAAGGTCAGTGCTATAGAAATTC | 40904 | 0.42523034783247815 | No Hit |
| CTCTCTTTCTGGCCTGGAGGCTATCCAGCGTACTCCAAAGATTCAGGTTT | 40418 | 0.420177982561439 | No Hit |
| GGCATCAAAAGTGATGGACTGCCCATTGCTGGAGAAGACCTTCTCTCCTA | 38918 | 0.4045842625890961 | No Hit |
| AACAAGCACAAAGCCAAGAAAACCAACGATGCCAGATACTGAATTTCTAT | 38551 | 0.4007689991025294 | No Hit |
| ACCTCTCTCTGGGCTTCTATTTCGACCGCGATGATGTGGCTCTGGAAGGC | 38179 | 0.39690175654938836 | No Hit |
| ATCAAAGAAGAACATGTGATCATCCAGGCCGAGTTCTATCTGAATCCTGA | 38071 | 0.3957790087113797 | No Hit |
| GAAACCCAGACACATAGCAATTCAGGAAATTTGACTTTCCATTCTCTGCT | 37335 | 0.38812769011161674 | No Hit |
| TTACAAAAGCAGAATAAAAGCGAAAAGAAATGAAAATGTTACACTACATT | 36471 | 0.37914570740754716 | No Hit |
| TGCCATATCCACATGGAAAATCTCATCACCATCAAAGTCAAACATAAACT | 29868 | 0.3105021520892934 | No Hit |
| TACCACTGCCACCTTCTCCATCGGCTCCACTGGCCTCGTGGTGTATGACT | 28543 | 0.2967276994470571 | No Hit |
| CTTCATGATGTAGCAGCAGGTGCCAGGGGCTGGCTTGTAGGCGATCAGCA | 26148 | 0.2718297265578828 | No Hit |
| GGCAATGAGCGGTTCCGCTGCCCTGAGGCACTCTTCCAGCCTTCCTTCCT | 24183 | 0.2514019533941135 | No Hit |
| GTCCACGTCACACTTCATGATGGAGTTGAAGGTAGTTTCGTGGATGCCAC | 23420 | 0.24346994783484835 | No Hit |
| TGTGGATGAATCTTGAACTGAGTTCCACTTGTAAACTTCTTGTTTCTTGT | 23361 | 0.24285659484926955 | No Hit |
| GTTCCATATCAGAGCTGTGATCTTGAGAGCCCTCTCCTTGGCTTTCCTGC | 21584 | 0.22438323458870055 | No Hit |
| CAGCATGTTCCCTCTCCTCATGAGATTGGTGAAGAAAGTATTTGGCAAAG | 20583 | 0.21397702546049036 | No Hit |
| ACGTTTACCTGTCCATGTCTTACTACTTTGACCGCGATGATGTGGCTTTG | 20203 | 0.21002661640083012 | No Hit |
| TCAGGAGACGCTCGTAGCCCTCGCGCTTCTCCTCGGCCAATTCGCGGAAG | 20111 | 0.20907020157585973 | No Hit |
| ACAAGAAGTACATCAAAGATTACATGAAATCAATCAAAGGGAAACTTGAA | 19381 | 0.2014812578559862 | No Hit |
| TCAGGAGACGCTCGTAGCCCTCGCGCTTCTCCTCGGCCAGTTCGCGGAAG | 18588 | 0.1932373778972742 | No Hit |
| GACGAGTCAGAAAGGTCACGTCCTGCTCTTGGTCCTTGTCAGTGCCATGT | 17747 | 0.18449449889944722 | No Hit |
| GTTCTGCAGCCCCTGTCATAAAAGGTTTTACTCTTTCTGGTCTCTGTTCT | 17461 | 0.18152129629138716 | No Hit |
| CAATAAATAGGACACTTCTGCCAAAGGAACTCGTGCACAGCACCACAGAA | 16925 | 0.17594914035460327 | No Hit |
| TTCAGCCCTGATCAAGACATGAGGGAGGCAGGGGCTCAGCTGAAGAAGCT | 16837 | 0.17503430878289247 | No Hit |
| GCCGACAAGACCAACGTCAAGGCCGCCTGGGGTAAGGTCGGCGCGCACGC | 16382 | 0.17030421372461513 | No Hit |
| GGCATCAAAAGTGATGGACTGCCCATTGCTAGAGAAGACCTTCTCTCCTA | 16166 | 0.1680587180485977 | No Hit |
| GCGTCTCTGTCTTCTTCAGTTTCGACTTATCGAATTTCTCGATCTCAGCC | 15983 | 0.1661562842119719 | No Hit |
| ACAAACGCGGCATAAGTTGACACATGGTCCGCCTTGATGGCCCCAGCTCC | 15519 | 0.16133262683386046 | No Hit |
| GGAAAGGGGGCACTGACTGGTGATGCTGATATTGGAGTGCATCTGAGGCT | 14313 | 0.14879527597609668 | No Hit |
| AGGTCTTGGTGGTGGGGAAGGACAGGAACATCCTCTCCAGGGCCTCCGCA | 14241 | 0.14804677741742422 | No Hit |
| TCTTGGGTTATAATCATTGGCAATGTTGTAATCAACAACCCATGTTATTT | 14178 | 0.14739184117858584 | No Hit |
| CGATCAGCACGGGCTCGTACTCTGCCACAAACTGATCACACTGCTTCTGG | 13968 | 0.14520872038245783 | No Hit |
| TCGCTCGTACTCGTGCGCCTCGCTTTGCTTTTCCTCCGCAACCATGTCTG | 13411 | 0.13941825236606112 | No Hit |
| GTCTCAACATCTGTGTTGGGGAGAGTGGAGACAGACTGACGCGAGCAGCC | 13348 | 0.13876331612722273 | No Hit |
| TTGGAGTGGGAAGCGGGGAATGTCTACGTGCGTATGCACGTGGCACTCTC | 13077 | 0.13594605071888607 | No Hit |
| TTGACTATACAACCAAAAAAGTTGCTGGATGTTTCTTTGACTACTGGAAC | 12618 | 0.13117437240734914 | No Hit |
| CGTGAATACCAAGACCTGCTCAATGTTAAGATGGCCCTTGACATTGAGAT | 12556 | 0.1305298319818256 | No Hit |
| GGAGAAAAACAGCACCAAGCAGGAGATCCTGGCTGCTCTTGAGAAAGGCT | 12523 | 0.1301867701424341 | No Hit |
| GAAAGGTGTGAGTTGGCCAGAACTCTGAAAAGATTGGGAATGGATGGCTA | 12467 | 0.12960460459679995 | No Hit |
| CAATGGGGTACTTCAGGGTCAGGATGCCACGCTTGCTCTGGGCCTCGTCG | 12467 | 0.12960460459679995 | No Hit |
| GTCCACGTCACACTTCATGATGGAGTTGAAGGTGGTCTCGTGGATGCCGC | 11667 | 0.12128795394488368 | No Hit |
| GGCAATGAGCGGTTCCGGTGTCCGGAGGCGCTGTTCCAGCCTTCCTTCCT | 11575 | 0.12033153911991332 | No Hit |
| TTTTTCCATGAGCTTAATGATGCTTTCTCTGGGCTTTTGGGGGAGGGTGT | 11527 | 0.11983254008079834 | No Hit |
| CTGACAGTGTATCTAGCTTTGGAAAACACAGGGGTCTGCCCTGTGAGCTG | 11095 | 0.11534154872876357 | No Hit |
| GGCAGACAAACCAGACATGGGGGAAATCGCCAGCTTCGATAAGGCCAAGC | 10842 | 0.11271140796009504 | No Hit |
| CAGGGAGGAAAAGTTTGGAAGAGGCAGAGAAATCCTGCTCTCCTCGCCTT | 10563 | 0.10981097604523926 | No Hit |
| CGAAGCGGTTGTACTTGCGGATGTAGTGCAGATAGTCTCGGCGGATGACA | 10167 | 0.1056942339725407 | No Hit |

## Adapter Content

Produced by FastQC (version 0.11.7)
